# Inferring the mode and strength of ongoing selection

**DOI:** 10.1101/gr.276386.121

**Published:** 2023-04

**Authors:** Gustavo V. Barroso, Kirk E. Lohmueller

**Affiliations:** Department of Ecology and Evolutionary Biology, University of California, Los Angeles, California 90095-1606, USA; Department of Human Genetics, David Geffen School of Medicine, University of California, Los Angeles, California 90095, USA

## Abstract

Genome sequence data are no longer scarce. The UK Biobank alone comprises 200,000 individual genomes, with more on the way, leading the field of human genetics toward sequencing entire populations. Within the next decades, other model organisms will follow suit, especially domesticated species such as crops and livestock. Having sequences from most individuals in a population will present new challenges for using these data to improve health and agriculture in the pursuit of a sustainable future. Existing population genetic methods are designed to model hundreds of randomly sampled sequences but are not optimized for extracting the information contained in the larger and richer data sets that are beginning to emerge, with thousands of closely related individuals. Here we develop a new method called trio-based inference of dominance and selection (TIDES) that uses data from tens of thousands of family trios to make inferences about natural selection acting in a single generation. TIDES further improves on the state of the art by making no assumptions regarding demography, linkage, or dominance. We discuss how our method paves the way for studying natural selection from new angles.

Genetic variation in natural populations is influenced by mutation, genetic drift, and natural selection. The fraction of mutations in genomes that are deleterious, neutral, or beneficial has been debated throughout the history of population genetics and remains controversial (Hey 1999; Kern and Hahn 2018; Jensen et al. 2019). One challenge is that genetic diversity from natural populations likely has been affected by multiple evolutionary forces simultaneously, making it hard to isolate and test for the effects of any one such force (Barroso and Dutheil 2021). However, many nonsynonymous mutations are detrimental (Keightley and Lynch 2003), and because natural selection has limited efficiency in removing them from the population, segregation of deleterious polymorphism is unavoidable, with direct consequences for the fitness of individuals (Agrawal and Whitlock 2011). In humans, for example, such genetic load contributes to an ∼50% miscarriage rate (Rice 2018), most of which occur in the first 12 wk of pregnancy. Inferring the strength of selection acting on these mutations (i.e., by how much they affect fitness of homozygous carriers, measured by the selection coefficient *s*) is therefore a central goal in biology (Eyre-Walker and Keightley 2007; Nielsen et al. 2007). Equally important is characterizing the degree to which new mutations influence fitness when counterbalanced by the presence of the “ancestral” allele in the homologous chromosome copy (i.e., their contribution to fitness of heterozygous carriers, measured by the dominance coefficient *h* in the canonical fitness function 1 + *h* × *s*) (Agrawal and Whitlock 2011; Huber et al. 2018). Together, knowledge of the selection coefficient (*s*) and the dominance coefficient (*h*) can inform about functional constraints in protein structure (Moutinho et al. 2019) and interaction networks (Park et al. 2019; Ratnakumar et al. 2020), shedding light into both evolutionary and medical genetics.

Existing methods to infer the strength of selection use the distribution of allele frequencies of a population known as the site frequency spectrum (SFS) to infer a distribution of fitness effects (DFE) of new mutations (Eyre-Walker et al. 2006; Keightley and Eyre-Walker 2007; Boyko et al. 2008; Kim et al. 2017; Tataru et al. 2017). Although they have greatly contributed to advancing population genetics in the past 15 yr (Moutinho et al. 2020), these models have important shortcomings, stemming mostly from the limited amount of information retained in the SFS. First, they treat selected sites independently, neglecting linkage disequilibrium (LD) (Slatkin 2008) and selective interference among them (Hill and Robertson 1966; Garcia and Lohmueller 2021). Second, they only incorporate oversimplified demographic histories, which are likely to be insufficient to accurately capture the effect of ancestral population sizes on genetic diversity. Third, because the equilibrium allele frequencies depend heavily on the fitness of heterozygotes, which in turn depend on the product of *s* and *h* but not on their individual values, the SFS alone cannot disentangle between these two crucial parameters (Huber et al. 2018). Consequently, the DFE is typically inferred assuming additivity; that is, the fitness of heterozygotes is the average of both homozygotes. This is problematic because deleterious mutations tend to be recessive, with heterozygotes having fitness values closer to those of individuals that are homozygous for the ancestral allele (Bosse et al. 2019; Huber et al. 2018). Fourth, and perhaps most importantly, the magnitude of selection can change over time (Orr and Betancourt 2001; Wittmann et al. 2017). For example, genes that have been highly constrained in the past may experience relaxation upon environmental change or even become more prone to mutations favored by selection. Conversely, previously neutral alleles may become deleterious. Several methods model a DFE with a proportion of positively selected variants (Boyko et al. 2008; Schneider et al. 2011; Galtier 2016; Zhen et al. 2021), but these methods still capture long-term signals of selection from the SFS, effectively averaging *s* over several thousand generations. Such averaging may result in misleading inference if there have been substantial fluctuations in selective pressures during the history of the population under study. Although progress has been recently made in inferring temporal trajectories of selection using ancient DNA samples (Mathieson 2020), a sufficient number of ancient samples is not always available from the relevant population. Finally, a different approach to find signatures of ongoing selection is to look for allele frequency changes with the age of present-day individuals (Mostafavi et al. 2017) or transmission distortions in family trios (Meyer et al. 2012). Both of these methods focus on finding variants having large effects on viability (by testing the null hypothesis of neutrality); however, they do not estimate selection or dominance coefficients, which are critical parameters to quantify the functional constraint of mutations. Taken together, these limitations reduce the ability of existing methods to accurately infer parameters of natural selection occurring in contemporary populations.

Here we suggest a way forward to overcome these challenges in inferring fitness effects for mutations that takes a different perspective of modeling natural selection acting on a single generation, rather than throughout the entire history of the population. Our new approach is made possible by the explosion of genome resequencing data from natural populations. For example, deCODE Genetics has genotype data on more than 160,000 individuals and 60,000 whole-genome sequences of much of the population of Iceland, including 2926 family trios (Halldorsson et al. 2019). Other projects like the UK Biobank have genotype data on 500,000 individuals, 200,000 exome sequences, and 150,000 whole-genome sequences (Halldorsson et al. 2022), whereas the TopMed project has sequenced 53,835 (Taliun et al. 2021), including 1465 family trios (Kessler et al. 2020). It is anticipated that within the next few years, entire populations will be sequenced, naturally incorporating hundreds of thousands of parent–offspring trios.

The availability of sequences from thousands of closely related individuals presents both an opportunity and a challenge. On the one hand, these data will provide an opportunity to overcome the aforementioned limitations of SFS-based methods in the inference of selection. On the other hand, they present methodological challenges, as traditional assumptions (e.g., absence of relatedness among sampled individuals) break down with such large samples. Further, such large data sets require dedicated statistical models that are accurate and computationally efficient. Here we overcome these challenges by developing a new model called trio-based inference of dominance and selection (TIDES) that is able to infer dominance and natural selection using tens of thousands of parent–offspring trios. Our method is designed to handle such large data sets efficiently, in anticipation of their availability in the near future. Moreover, a unique feature of TIDES is that it is sensitive to the strength of selection acting on the current generation, and it is therefore ideal to study population-specific signatures of selection while not being confounded by other evolutionary forces like demography or like averaging selective effects over long time periods. TIDES can be applied to either sets of variants across the genome or a single variant at a time, further showcasing its flexibility.

## Results

### Overview of the model

**Figure 1.**
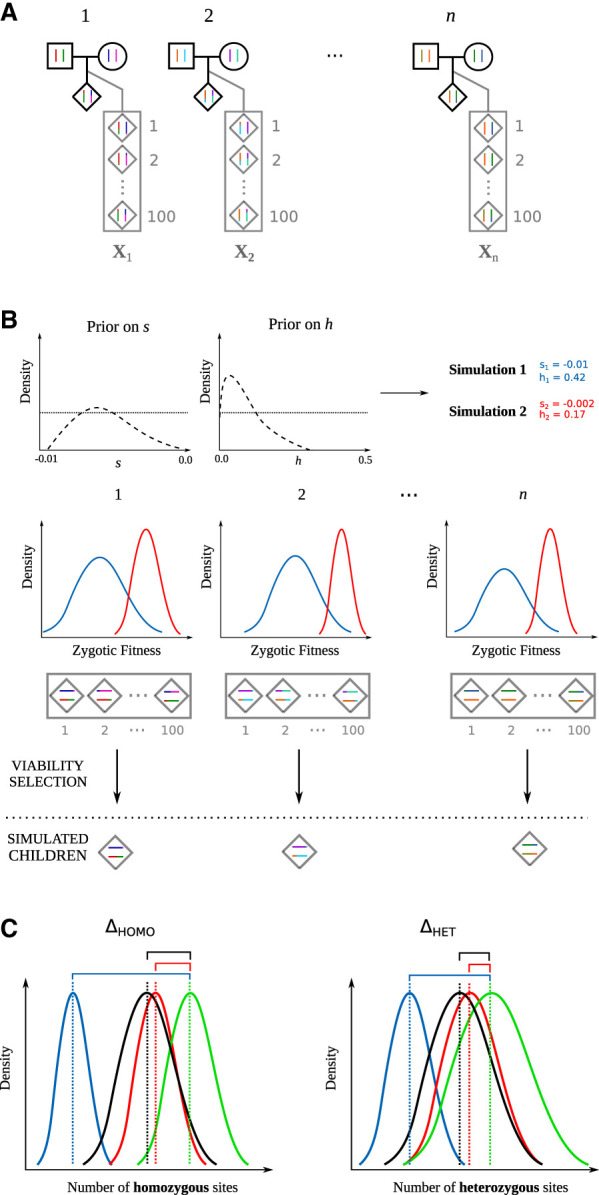
Schematic representation of TIDES. (*A*) Observed family trios (black outline) together with the zygotes generated from parental haplotypes (gray outline). (*B*) Illustration of the TIDES simulation engine for two draws from the prior distribution of *s* and *h*, representing strong (blue) and weak (red) values of selection. The *middle* row shows the computation of zygotic fitness and natural selection. (*C*) Comparison between the observed summary statistics (black offspring/green parents) and the summary statistics from the simulations using the selection parameters from the prior distribution (red and blue). The *left* panel shows the comparison of the number of homozygous genotypes (Δ_HOMO_), and the *right* panel shows the comparison for the number of heterozygous genotypes (Δ_HET_). In this example, the values of *s* and *h* from the red parameter combination better fit the observed data than do the parameters shown in blue.

TIDES uses approximate Bayesian computation (ABC) (Pritchard et al. 1999; Beaumont et al. 2002; Beaumont 2019) to model the effect of selection on genetic diversity during the span of a single generation. It leverages phased sequences from parent–offspring trios to detect signatures of selection in the transmission of single-nucleotide polymorphisms (SNPs) (Meyer et al. 2012) and uses this information to infer dominance and selection coefficients. By exploiting the random shuffling of haplotypes during meiosis, family trio data become immune to external confounding factors that lurk in traditional population genetic studies (Bates et al. 2020), such as nonequilibrium demography (Sul et al. 2018; Barton et al. 2019). The first step in our simulation framework is to use parental haplotypes and recombination maps to generate an array of potential zygotes for each trio (note that de novo mutations are identified and removed from children, because they are not transmitted from the parents) (Fig. 1A; Algorithm 1). We then sequentially impose rounds of viability selection on the simulated zygotes, for independent values of *s* and *h* drawn from their prior distributions, and compute summary statistics from the set of “selected” zygotes (Fig. 1B). Comparing genomes from children (the observed data) with genomes from simulated zygotes that could have been conceived by their parents provides information about the (unobserved) embryos that did not survive and is therefore indicative of the strength of selection.

In essence, TIDES is a fitness-based model that mimics the process of meiosis followed by natural selection. We compute the fitness *f* of each individual (both real and simulated) using a multiplicative model:

(1) $$f={\left(1+s\right)}^{\left[k_{\mathrm{homo}}\right]}\times {\left(1+h\times s\right)}^{\left[k_{\mathrm{het}}\right]},$$

where *k*_homo_ and *k*_het_ are the counts of derived sites in homozygous (i.e., carrying two copies of the mutant allele) and heterozygous states, respectively. To compute summary statistics, we consider the combined genetic diversity of the parents separately from the combined genetic diversity of the offspring. We use the relative differences in the averages of *k*_homo_ and *k*_het_ between offspring and parents as our two summary statistics, denoted Δ_homo_ and Δ_het_. Specifically, let *x*_0_, *y*_0_ be the average number of homozygous and heterozygous sites among the entire parental generation, and *x*_1_, *y*_1_ be the corresponding averages among children. We define *Q*_obs_ as the vector storing the quantities Δ_homo_ = *x*_1_ – *x*_0_ and Δ_het_ = *y*_1_ – *y*_0_. Likewise, if *x*^(*i*)^_2_ and *y*^(*i*)^_2_ are the averages among simulated zygotes that have survived selection for the *i*th parameter combination, then $Q_{\mathrm{sim}}^{\left(i\right)}$ is the vector storing $\Delta_{\mathrm{homo}}=x_{2}^{\left(i\right)}-x_{0}$ and $\Delta_{h\mathrm{et}}=y_{2}^{\left(i\right)}-y_{0}$. Disregarding the influx of de novo mutations (which could balance the effect of selection on genetic diversity), such trio-based summary statistics can be used to model both negative and positive selection on a set of candidate sites. When modeling negative selection, higher values of |*s*| (corresponding to stronger negative selection) should lead to sharper reductions in the overall number of deleterious variants in the offspring and therefore lower values of both Δ_homo_ and Δ_het_. Higher values of *h* should result in lower values of Δ_het_ but not Δ_homo_. Conversely, when modeling positive selection, higher values of *s* (corresponding to stronger positive selection) should lead to sharper increases in the overall number of beneficial variants in the offspring and therefore higher values of both Δ_homo_ and Δ_het_. Higher values of *h* should result in higher values of Δ_het_ but not Δ_homo_. Therefore, retaining genotype information instead of reducing genetic diversity to the SFS is key to disentangling the combined effects of dominance and selection.

### Evaluating the performance of TIDES

#### Inferring the exome-wide strength of negative selection

We first evaluate TIDES’ ability to infer the strength of negative selection on a set of deleterious SNPs. To benchmark our model and method, we simulated trio sequence data from a population reflecting the European demographic history (Gravel et al. 2011) and sex-specific recombination maps (Halldorsson et al. 2019), as well as the human exome structure using SLiM (Methods) (Haller and Messer 2019). These simulated data should reflect a reasonable picture of deleterious standing variation, in terms of both derived allele frequencies and their LD patterns. We then sampled 60,000 individuals from the final generation, matched females and males at random to generate offspring, and finally down-sampled to 50,000 trios in each scenario, which became our test data sets for inference. Throughout the following simulation study, we used flat, uninformative priors in order to assess TIDES' ability to extract information from the data when there is weak a priori knowledge about the parameters. Specifically, we let *h* be uniformly distributed in the open interval (−0.1; 0.6) and *s* be log-uniformly distributed in the open interval (−10^−5^; −10^−1^) (except for neutral simulations, where *s* was uniformly distributed in the open interval (−0.01; 0.01)).

**Figure 2.**
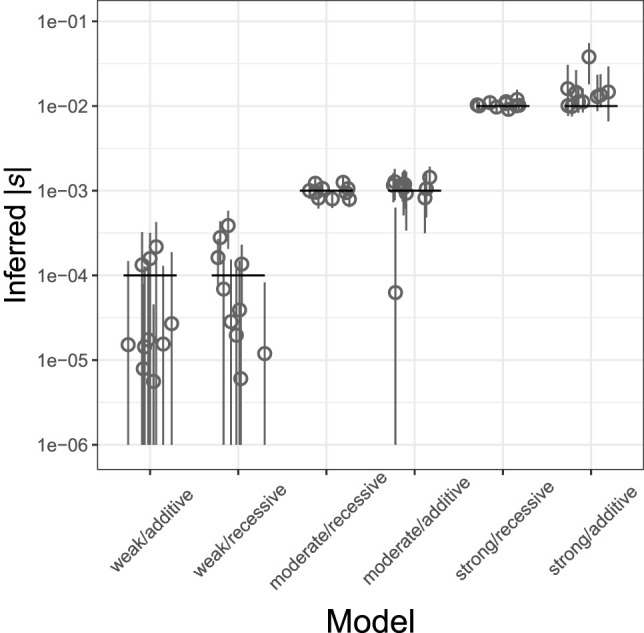
Inference of *s* from a genome-wide set of deleterious SNPs for different strengths of selection (weak: *s* = −0.0001; moderate: *s* = −0.001; strong: *s* = −0.01) and dominance effects (recessive: *h* = 0; additive: *h* = 0.5). Each scenario includes the estimates from 10 simulated data sets. True values are shown as black horizontal segments, with medians of the inferred posterior distributions denoted by gray circles and their 95% credible intervals by gray vertical lines. The *y*-axis is in log_10_ scale; all values are in absolute numbers. Here, 50,000 trios are used.

We found that in neutral simulations, TIDES infers *s* to be centered around zero, and in all replicates, the posterior distributions include both positive and negative values (Supplemental Fig. S1), indicating that noise in the sampling of parental SNPs (i.e., genetic drift) does not generate a spurious signal of selection. TIDES has overall high accuracy in the six combinations of *s* (−10^−4^, −10^−3^, −10^−2^) and *h* (0, 0.5) that we tested, with the medians of the inferred posterior distributions centered around the true values of *s* (Fig. 2). In general, accuracy is higher in the recessive scenarios and especially as selection becomes stronger. The small difference in power between additive and recessive scenarios is reflected as a negative correlation between posterior samples of *s* and *h* in some additive simulation replicates (Supplemental Fig. S2). The large difference in power with stronger selection occurs because we treat our sample of parents as a de facto population and project it forward by one generation, where (disregarding de novo mutations) negative selection has the opportunity to reduce deleterious genetic diversity. Therefore, similarly to the classic population genetics result in which selection becomes more efficient as |*N*_e_ * *s|* grows beyond one (Kimura 1979), the product of *n* (the number of trios) and |*s|* must be large enough for TIDES to have high inferential power. Indeed, for our sample size of 50,000 trios, |*n* * *s|* equals 500 in the strong selection scenario and 50 in the moderate selection scenario, but equals only five when selection is weak. Taken together, these results suggest that for sufficiently large sample sizes, our trio-based framework can accurately infer the strength of ongoing selection for an arbitrary range of selection coefficients.

**Figure 3.**
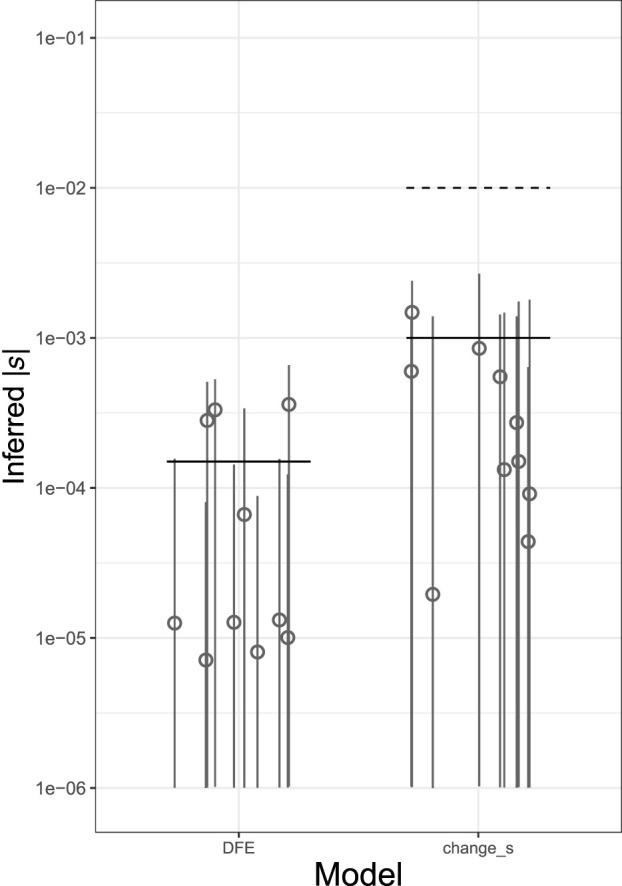
Inference of *s* from a genome-wide set of deleterious SNPs under complex models of selection. Each scenario includes the estimates from 10 simulated data sets. *Left* panel shows the results when the true DFE follows a gamma-distribution (mean value of *s* shown by black horizontal line). The *right* panel shows the case in which there was a 10-fold reduction in the selection coefficient. Ancient and current values of *s* shown by dashed and solid horizontal lines, respectively. Medians of the inferred posterior distributions denoted by gray circles and their 95% credible intervals by gray vertical lines. The *y*-axis is in log_10_ scale; all values are in absolute numbers. Here, 50,000 trios are used.

It has been well established that the selection coefficients of deleterious SNPs vary widely, from nearly neutral to lethal (Eyre-Walker et al. 2006; Boyko et al. 2008; Kim et al. 2017). To assess the performance of TIDES in the presence of a DFE, we performed simulations in which the selection coefficient of new mutations comes from a gamma-distributed DFE parameterized by α = −0.186 and β = 0.071. As negative selection purges strongly deleterious alleles more efficiently, the average selection coefficient of segregating SNPs is expected to be less negative than that of new mutations. In our simulations of European evolutionary history, the average *s* of new mutations is −0.013, whereas that of segregating SNPs is −0.00015. Because TIDES focuses on a single generation instead of modeling long-term frequency trajectories, it infers the average *s* of SNPs segregating in the parents. Although the value of −0.00015 falls within the weak selection regime, where our power with 50,000 trios is reduced, the medians of the posterior distributions are located near the true value (Fig. 3), showing that when mutations have different selection coefficients, TIDES can infer their average.

Finally, we asked whether TIDES could capture a shift in the strength of selection happening in current generation. The goal of this simulation was to assess the performance of our method in a scenario of environmental change in which the selective pressure is abruptly reduced. To this end, we started from the same parental sequences from the recessive scenario with historical *s* = −10^−2^, except this time we imposed viability selection in the offspring generation by changing *s* of all segregating SNPs to −10^−3^. Although a 10-fold decrease in the strength of selection is a statistically challenging signal to capture (because the level of standing variation reflects the previously stronger selection and is therefore reduced relative to the new expectation, with respect to both the count of deleterious SNPs as well as their frequencies), TIDES recovers the updated value of *s* (Fig. 3), showcasing that our model is sensitive to the strength of ongoing selection and is not burdened by memory of the past.

#### Inferring the selection coefficient of a single deleterious SNP

**Figure 4.**
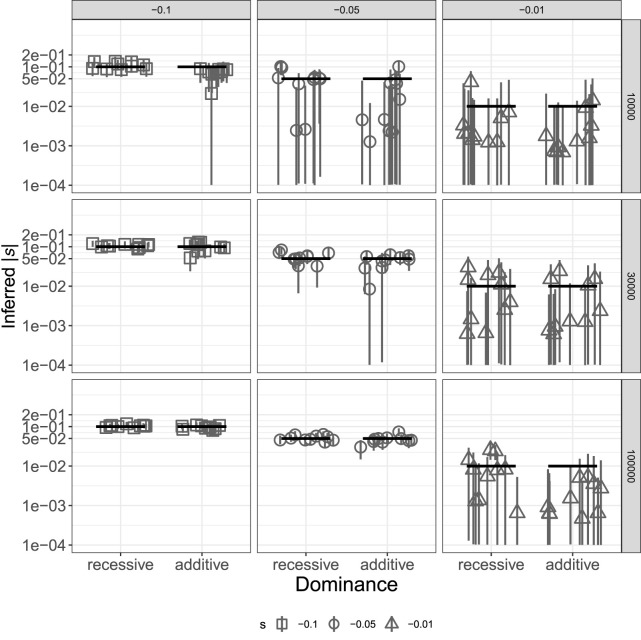
Inference of *s* for a single deleterious SNP with different dominance effects. Each scenario includes the estimates from 10 simulated data sets. Columns show different values of the true selection coefficient, and rows show different sample sizes, in terms of the number of trios used. True values are shown as black horizontal segments, with medians of the inferred posterior distributions denoted by gray shapes and their 95% credible intervals by gray vertical lines. The *y*-axis is in log_10_ scale; all values are in absolute numbers.

The results above suggest that TIDES can infer the (average) selection coefficient from a set of deleterious SNPs, but in some situations, single SNPs may be of interest. A few methods have been recently developed to infer selection coefficients of single SNPs using contemporary data (Stern et al. 2019) or their temporal trajectories using ancient DNA data (Mathieson 2020), but tools for detecting ongoing selection in the focal population are still lacking. To achieve high accuracy with single variants in TIDES, we once again should require that |*n* * *s*| >> 1, noting that only informative trios (those where at least one parent is heterozygous, hence the couple has the potential to produce more than one offspring genotype) should be included in the analysis. Because interest in individual SNPs may be motivated by situations in which large effects are expected, we tested TIDES' accuracy to infer strong negative selection using the open interval (−10^−4^; −10^0^) as a log-uniform prior on *s*. We simulated data sets where the frequency *q* of the deleterious allele among parents is ∼0.5, and we varied the number of informative trios (10,000, 30,000, or 100,000), as well as *s* (−0.01, −0.05, or −0.1) and *h* (0.0 or 0.5). TIDES is accurate in all scenarios of *s* = −0.1, whereas it requires at least 30,000 informative trios to have high accuracy when *s* = −0.05 and does not start to perform well until 100,000 trios and recessive selection for *s* = −0.01 (Fig. 4). TIDES’ accuracy on single SNPs depends on higher values of |*n* * *s|* than for exome-wide inference (because all couples are highly informative in the latter case owing to the large number of SNPs they carry), but given enough data, our model is able to infer ongoing selection on a single deleterious variant.

In genomic regions with unusually strong and positive LD, however, TIDES’ inference on a single deleterious SNP could potentially be biased by transmission distortion induced by the joint effect of neighboring SNPs. To assess the magnitude of this effect, we simulated an extreme scenario in which 11 SNPs are 1 kb apart from each other with pairwise *D*′ values all equal to one (i.e., maximum LD between each SNP) and the recombination rate per site in the generation of sampling is 10^−12^ (i.e., 10,000 times lower than the genome-wide average in humans). We then used TIDES to infer *s* and *h* on the middle SNP (sixth position in the sequence) and ignored the presence of its 10 constrained neighbors during the inference. Using 10,000 trios, we found that when selection on the focal SNP is 10 times stronger than selection on each of the neighboring SNPs (*s* = −0.1 vs. −0.01), our inference remains accurate (Supplemental Fig. S3A). However, if selection is equally strong against all 11 variants (*s* = −0.1), the other linked deleterious variants bias the inferences of selection at the focal variant (Supplemental Fig. S3A). This is expected as the fully linked SNPs reduce fitness and contribute to the inferred value of *s*, but are not being modeled in the inference framework. The effect of hidden linkage on the inference of *h* is more complex, with 11 equally deleterious variants leading to a small upward bias but substantially reduced variance of the posterior distributions (Supplemental Fig. S3B). Because SNPs of similar deleterious effects will tend to be found in negative as opposed to strongly positive LD (Hill and Robertson 1966; Garcia and Lohmueller 2021), the scenario we simulated aimed primarily to test the technical limitations of our method, but is not anticipated to occur in real data sets. However, caution should be taken when using TIDES on single SNPs that belong to tight linkage blocks. In these cases, we recommend also inferring parameters using all the SNPs in the region and comparing the posterior distributions from both procedures.

Because the frequency of deleterious SNPs segregating in natural populations is inversely proportional to the strength of negative selection against them, more strongly deleterious variants will tend to be kept at a lower frequency, requiring larger samples from the population in order to find a sufficient number of informative trios. On the other hand, the number of informative trios required for inference decreases with increasing deleteriousness of variants because, in this case, each SNP exerts a stronger signal in the data (i.e., sharper transmission distortion). Therefore, the overall sample size required for accurate inference (with subsequent down-sampling to consider only informative trios) is a function of both of these parameters that act in opposing directions. To assess whether it is realistic to expect that any single, strongly deleterious, variant segregates at appreciable frequencies in humans, we performed simulations with exponential population growth in which the final population size is 10,000,000 and assumed that the total number of target sites subject to mutations of each selection coefficient is 1,000,000 for *s* = −0.1, 5,000,000 for *s* = −0.05, and 5,000,000 for *s* = −0.01 (Methods). In all cases, we found SNPs segregating at the absolute frequency thresholds required for accurate inference with TIDES (Table 1). In other words, the simulations show that we would expect a few strongly deleterious SNPs to segregate at frequencies high enough such that it would be possible to subset a collection of 10,000,000 total trios down to a sample size where both *q* ∼ 0.5 and we meet the required number of informative trios. We conclude that there are strongly deleterious variants likely segregating in the population at sufficient frequency to be analyzed using TIDES in the foreseeable future. With the sample sizes described here, it will already be possible to use the single-variant model in TIDES to test for strong ongoing selection, which can provide valuable biological information for prioritizing particular sites of functional importance (e.g., in regulatory regions).

**Table 1.**
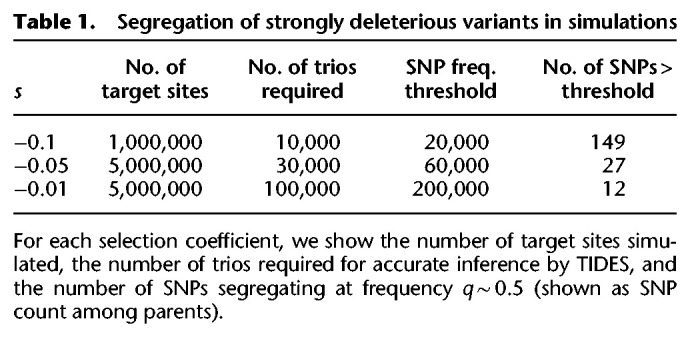
Segregation of strongly deleterious variants in simulations

#### Inferring the selection coefficient of a single beneficial SNP

**Figure 5.**
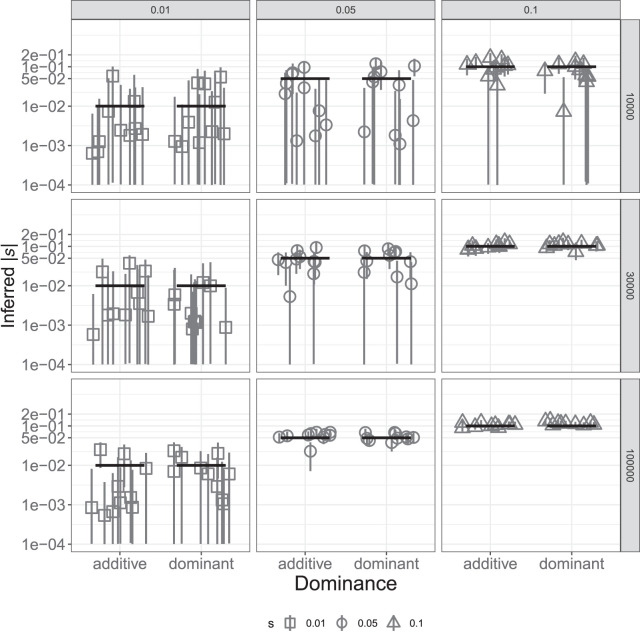
Inference of *s* from a single beneficial SNP with different dominance effects. Each scenario includes the estimates from 10 simulated data sets. Columns show different values of the true selection coefficient, and rows show different sample sizes, in terms of the number of trios used. True values are shown as black horizontal segments, with medians of the inferred posterior distributions denoted by gray shapes and their 95% credible intervals by gray vertical lines. The *y*-axis is in log_10_ scale.

Although strongly deleterious SNPs tend to segregate in very small numbers even in large samples, the opposite is true for beneficial variants subject to positive selection. Although the fraction of the genome in which mutations are expected to be beneficial is considerably smaller than that for deleterious mutations, there are examples of SNPs putatively under strong positive selection in recent human history (Stern et al. 2019; Mathieson 2020). To investigate whether TIDES can infer the strength of positive selection acting on single SNPs, we simulated data sets in which the frequency *q* of the beneficial allele among parents is ∼0.5, and we varied the number of informative trios (10,000, 30,000, or 100,000), as well as *s* (0.01, 0.05, or 0.1) and *h* (0.5 or 1.0). We opted for including fully dominant beneficial alleles in order to assess TIDES' power in this typically less explored (but still plausible) scenario. When performing inference, we flipped the sign of the prior on *s* (log-uniform in the open interval (10^−4^; 10^0^)). As expected, our statistical power is very similar to the analogous analysis of negative selection. TIDES is accurate in all scenarios of *s* = 0.1, whereas it requires 30,000 informative trios to have high accuracy when *s* = 0.05 and does not start to perform well until 100,000 trios and dominant selection for *s* = 0.01 (Fig. 5). Because such variants are expected to segregate at a range of frequencies on their way to fixation (depending on their age and fitness effect), finding the necessary number of informative trios should not require that entire populations are sequenced, expediting the application of TIDES to study candidate single beneficial SNPs. A particularly attractive application of our method will be to test whether variants with a strong signal of positive selection in the past few thousand years of human evolution are still under strong selection today. Lactase persistence is a typical example of such recent and strong positive selection (Bersaglieri et al. 2004), with the 13910*T variant segregating at frequencies of ∼0.77 and ∼0.43 in Northern and Southern Europeans, respectively (Liebert et al. 2017). Therefore, a total of approximately 40,000 random trios from Northern Europe or approximately 25,000 random trios from Southern Europe would be required to find approximately 10,000 informative trios in each of these populations. Assuming that the children are old enough such that the advantage conferred by the derived allele (if any) has had the opportunity to be manifested, with these numbers, it will already be possible to test the hypothesis that the strength of (population-specific) ongoing selection is ∼0.1 or greater for this allele.

#### Inferring the dominance coefficient

**Figure 6.**
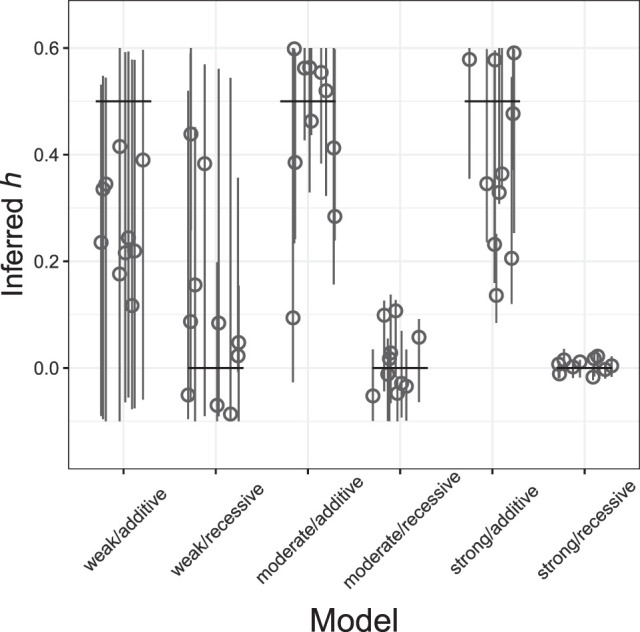
Inference of *h* from a genome-wide set of deleterious SNPs for different strengths of selection (weak: *s* = −0.0001; moderate: *s* = −0.001; strong: *s* = −0.01) and dominance effects (recessive: *h* = 0; additive: *h* = 0.5). Each scenario includes the estimates from 10 simulated data sets. True values are shown as black horizontal segments, with medians of the inferred posterior distributions denoted by gray circles and their 95% credible intervals by gray vertical lines. Each scenario includes 50,000 trios.

Modeling the transmission of genotype counts in family trios allows us to tease apart the selection coefficient *s* from the dominance coefficient *h* because *h* directly impacts the expected number of heterozygous but not homozygous sites in the children (Eq. 1). When inferring posterior distributions for the dominance coefficient using the exome-wide data from the simulations described above, we observe similar trends in accuracy as for the selection coefficient: The posterior distributions fall near the true values of *h* in both the strong and moderate selection scenarios, but not for weak selection, because the small value of |*n* * *s|* also affects our ability to infer *h* from SNP transmission distortions (Fig. 6; Supplemental Fig. S2). On the other hand, estimating the dominance coefficient for single variants is more challenging than estimating the selection coefficient in terms of the required sample size. For both negative selection (Supplemental Fig. S4) and positive selection (Supplemental Fig. S5), posterior distributions of *h* are too wide when |*s*| = 0.01 (spanning almost the entire range of the prior), whereas we need more than 30,000 informative trios when |*s*| = 0.1 and more than 100,000 informative trios when |*s*| = 0.05 for accurate inference.

**Figure 7.**
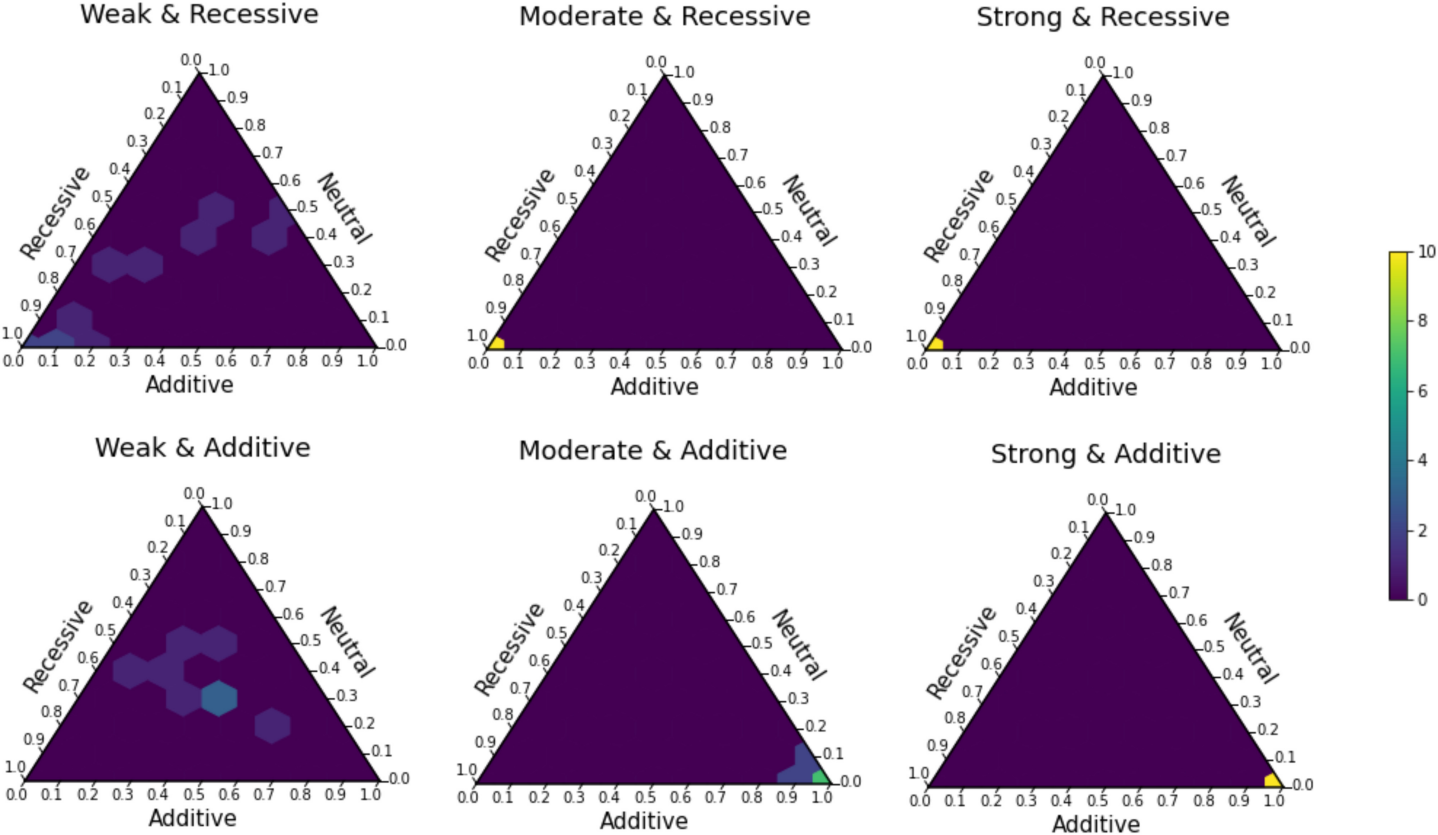
TIDES can accurately distinguish among neutral, recessive, and additive models of selection. True models are shown *above* each simplex (weak: *s* = −0.0001; moderate: *s* = −0.001; strong: *s* = −0.01; recessive: *h* = 0; additive: *h* = 0.5). The coordinates along each axis denote posterior probabilities assigned to the respective model. The color of each tile represents the proportion of simulations that fall within that probability bin (scale shown at *far right*).

In addition to inferring posterior distributions for *h*, we can compare the fit of different models of dominance through Bayesian model selection (Csilléry et al. 2010, 2012). To this end, we fitted constrained models to each exome-wide data set and then computed posterior probabilities for each model based on their acceptance rates in the standard rejection algorithm. We tested TIDES’ ability to distinguish between “neutral,” “additive,” and “recessive” models. In the “additive” and “recessive” models, *h* is fixed to 0.5 and zero, respectively, and only *s* is drawn from its prior distribution. In the “neutral” model, *s* is fixed to zero, and any fluctuation in the frequency of SNPs is owing to genetic drift alone. To benchmark the accuracy of our method in model selection, we ascribed equal prior probabilities to the three models (in ABC, we do this by considering the same number of candidate simulations under each model). Using this framework, TIDES shows remarkable accuracy in classifying data sets (Fig. 7), with posterior probabilities of 1.0 being assigned to the correct model in all 20 replicates of the strong selection regime (*s* = −0.01). Likewise, posterior probabilities of 1.0 are assigned to “recessive” in all 10 replicates of recessive and moderate selection (*s* = −0.001), whereas posterior probabilities > 0.9 are assigned to “additive” in all the 10 replicates of additive and moderate selection. As seen above for quantitative parameter inference, the weak selection regime (*s* = −0.0001) is the most challenging, in which the posterior probabilities are diffuse across the three models in all 20 replicates. Here, TIDES often cannot reject a neutral model of evolution, in agreement with sample size being insufficient for efficient negative selection in the parents. In summary, besides inferring the strength of selection, our model offers high accuracy to distinguish between recessive and additive models of the genotype to fitness map.

## Discussion

Sequence data from family trios offer a fresh perspective for the inference of natural selection. We have implemented these ideas into a new statistical model called TIDES, which has several conceptual improvements over traditional SFS-based methods. First, the parent–offspring structure grants immunity to biases arising from a complex demographic history (Bates et al. 2020). Demography *sensu lato* (including population size changes, subdivision, and migration) has been shown to be a confounder in the inference of selection in general (Williamson et al. 2005; Nielsen et al. 2007, 2009), and eliminating this effect is considered a major enhancement in model design. Second, TIDES does not require the specification of putatively neutral variants for contrasting frequency spectra. Third, we explicitly model linkage and selective interference among SNPs, which improves estimates of *s* and *h* compared with a maximum likelihood approach that assumes SNPs are independent of one another (Supplemental Note S1; Supplemental Figs. S6–S9). Fourth, TIDES jointly infers the dominance coefficient *h.* This not only improves inference of *s* by integrating it over a range of dominance values but also enables directly testing for additive versus recessive effects of mutations, a notoriously challenging problem in human population genetics because different combinations of values for *s* and *h* can yield the same SFS. Moreover, methods to infer strong negative selection must rely on the assumption of mutation-selection balance (Haldane 1937; Weghorn et al. 2019) and hence can only infer the strength of selection against the heterozygote genotype (Cassa et al. 2017). Finally, TIDES is notably sensitive to the strength of selection acting on the current generation. This is a unique feature of our method that avoids conflating the selective constraint from different periods in time into a single estimate. For instance, it is conceivable that human cultural evolution (the advent of medicine in particular) may have modulated the selective pressure on several genes, and our method captures the updated state of selective constraint. These improvements come at the cost of targeting inference at the average *s* rather than a DFE (when using multiple SNPs) and requiring sample sizes of the order of tens of thousands of trios. Fortunately, extravagantly large genomic data sets are becoming commonplace, and we anticipate that TIDES will make its debut analyzing human data within the next few years. The application to domesticated species will follow, in which breeding and genetic engineering are used to impose changes along phenotypic gradients.

There are yet other properties that distinguish TIDES from existing methods. First, because TIDES computes summary statistics conditioning on the parental haplotypes, it is able to perform inference in biased data sets (e.g., in which genetic load is expected to be higher than average), whereas existing methods require random samples from the population. Second, the type of polymorphism analyzed is not restricted to SNPs. Using our ABC approach, it is straightforward to infer the *s* and *h* from structural variants such as copy number variation and chromosomal inversions, some of which have already been hypothesized to have strong phenotypic and fitness effects (Alonge et al. 2020; Hämälä et al. 2021). Third, the age of offspring at the moment of sampling is relevant to understand which components of fitness are being captured by the model (Orr 2009). In the simplest case of SNPs implicated solely in embryonic development, TIDES will infer parameters concerning viability selection acting up to the time of birth. Estimating *s* and *h* for SNPs whose effects are manifested later in life requires that offspring are sequenced after the desired phenotype has developed (and consequently its fitness effect has been realized; for a consideration of the case of the lactase persistence phenotype, see Results). A more complex picture arises for SNPs that influence viability throughout adulthood. In these cases, the deleterious effects of the SNPs on fitness can be modeled as increasing the probability of death per unit time throughout the individual's lifetime, such that the frequency of the deleterious alleles decreases with age in a stratified sample (Mostafavi et al. 2017). Incorporating the effect of this class of SNPs into our framework could involve, for example, an exponential decay of offspring survival as a function of both their fitness and their age, with different flavors of pleiotropy introducing further complexity. Modeling these nuances is a benefit of ABC and contrasts with methods that infer long-term selection, which conflate all fitness components (including relative fitness) into a single estimate. Considering the flexibility that results from TIDES’ properties put together, we anticipate that it will fundamentally change inference of natural selection.

The current implementation of TIDES uses the difference in the counts of heterozygous- and homozygous-derived genotypes between parents and offspring as the key summary of the data. A possible extension of TIDES could include additional summary statistics of genetic variation data, such as allele frequency information or patterns of LD. Although such richer statistics should extract additional information from the data, they come at a computational cost because of the additional bookkeeping needed to evaluate them at every simulation step. Another future direction could be to jointly leverage transmission patterns in trios with counts of heterozygous and homozygous genotypes in large samples of individuals from the population to coestimate *s* and *h*. Although this approach would have the advantage of leveraging large data sets of unrelated individuals, by using patterns of standing genetic variation, it would not be as robust to demographic processes, like population structure or assortative mating. Nevertheless, this is a promising avenue for future work.

A potential limitation of TIDES in real data applications is to distinguish the small effect of transmission distortion on the background of biases of short-read sequencing data if some of these biases correlate with parameters of selection. Specifically, genotyping errors can lead to over transmission of the major or reference allele in family-based association studies owing to undercalling heterozygous genotypes in the offspring (Mitchell et al. 2003; Yan et al. 2016). Such genotyping errors in TIDES could lead to an overestimation of the strength of selection as it would appear that the minor (putatively deleterious) alleles are being undertransmitted. Thus, data quality is of paramount importance for studies using transmission patterns, and only variants in the accessible part of the genome should be considered. Additionally, as a control analysis, TIDES could be applied to putatively neutral variants (i.e., intronic variants). If selection was detected, it would suggest that data artifacts could be driving transmission patterns. Nevertheless, despite these potential caveats, we are confident that this challenge will eventually be overcome as improved sequencing methods are being developed.

A thought-provoking possibility is that TIDES may open an avenue for experimental evolution in multicellular organisms with relatively long generation times. Our results suggest that it is possible to infer *s* and *h* of single SNPs artificially introduced in model species (e.g., by CRISPR) (Ran et al. 2013). By measuring fitness in a large sample, but in a single generation (as opposed to small samples collected over hundreds of generations, as traditionally performed in single-cell organisms), TIDES can be used to study fitness effects of mutations (e.g., Sarkisyan et al. 2016) in highly constrained genes in which natural variation is too low for traditional methods to work. Much like sequencing itself, the ease and cost of genetically editing model organisms are expected to greatly improve in the upcoming years, broadening the range of application of single-generation inference of selection.

TIDES has potential application to management of species of conservation concern. In managed populations, biologists may choose which individuals mate. The fitness model used in TIDES could be applied to aide in selecting which individuals likely have the lowest burden of deleterious mutations. Specifically, parents whose offspring have similar counts of heterozygous- and homozygous-derived genotypes as they do likely carry lower burdens of deleterious variation than do parents whose offspring have substantially lower counts of heterozygous- and homozygous-derived genotypes. Thus, such parents could be prioritized in controlled breeding programs. More generally, TIDES will help determine the dominance effects of deleterious mutations, which will aide in understanding the relative importance of strongly deleterious recessive mutations versus additive weakly deleterious mutations at reducing fitness in small populations.

Future progress in genomics depends on extracting information from large data sets in which assumptions about the relatedness of individuals (or lack thereof) break down. We have shown that going beyond random samples from a population allows statistical methods to capture signals that are both more subtle (e.g., with respect to timescale) and more robust (requiring fewer assumptions about the data-generating process) than the current state of the art. Therefore, we believe that upcoming studies should prioritize the inclusion of family trios or larger pedigrees in their sequencing efforts (Meyer et al. 2012) because they provide an overall richer data structure that can be exploited to infer present-day recombination rates (Halldorsson et al. 2019), mutation rates (Francioli et al. 2015), and now dominance as well as selection. As TIDES conditions on parental haplotypes, any new “child” in the data set will add a similar amount of information, regardless of its kinship coefficient with other members of the data set (siblings, *trans*-generational pedigrees, etc.). Thus, larger pedigrees may be used in TIDES without loss of accuracy. We hope that as the TIDES framework continues to develop, it will also inspire other groups to consider how to leverage the future abundance of family trio data to infer other types of selection.

## Methods

TIDES is a fast simulator of meiosis followed by selection. We opted to embed TIDES in an ABC framework because its high flexibility will foster extensions of the model in the future. Here we outline a typical execution with default options. For each of *n* trios, TIDES simulates an array of 150 zygotes. The number of zygotes each parent generates is proportional to the number of children they have in the data set, avoiding bias in the presence of siblings. Because meiosis is independent of *s* and *h*, the *n* arrays are preconstructed and retained throughout the execution of the program (Fig. 1A), substantially improving computational performance. TIDES then iteratively (1) draws *s* and *h* values from their prior distributions, (2) computes the fitness of all zygotes and samples once per trio with probability proportional to their fitness (Fig. 1B), and (3) computes summary statistics for the batch of “selected” zygotes (note that sampling one zygote with probability proportional to its fitness is more efficient than sampling one zygote at random, imposing viability selection, and repeating this process until a zygote survives in each trio) (Fig. 1C; Algorithm 1). Contrasting summary statistics computed from the simulated survivors (*Q*_sim_) with those computed from the actual children (*Q*_obs_) offers an objective approach to inference: Values of *s* and *h* that generate substantial differences between *Q*_sim_ and *Q*_obs_ are discarded, whereas those that best agree are used to paint posterior distributions for the parameters. In the pilot run, we use a uniform prior (e.g., (−0.1; 0.6)) for *h* and a log-uniform prior (e.g., (−10^−5^; −10^−1^)) for *s*, the latter in order to more frequently sample from regions of low selection coefficients that would otherwise not be sufficiently explored by a uniform prior.

Given sufficient summary statistics, the accuracy of ABC converges to that of full likelihood methods as the acceptance rate of proposed parameters decreases toward zero (Beaumont 2019). In practice, the performance of specific ABC methods increases with the total number of simulations performed (Csilléry et al. 2010). Therefore, motivated by improving computational efficiency, several approaches have been developed to sample parameter values from a region of high posterior density (Sisson and Fan 2018). These mostly rely on proposing a new set of values conditional on the acceptance of previous proposals. Consequently, these approaches complicate parallelization of the simulation engine, being of most value when individual simulations are computationally expensive. When focusing on the one-generation interval between parents and offspring, however, each individual simulation is computationally cheap such that, in TIDES, we prioritized multithreading over more elaborate sampling techniques. After obtaining a reference table of the simulation using TIDES, all downstream analyses regarding model selection and parameter inference were performed using a combination of the packages abc, rethinking, scales, cowplot, and tidyverse in R 3.6 (scripts available in the GitHub repository; see Software availability) (R Core Team 2020).

The algorithms below briefly summarize the ABC implementation of TIDES.

### Algorithm 1

1. *Setting up*
  Compute summary statistics *Q*_obs_ for the actual children;
  For each trio 1..*n*, generate 150 zygotes based on parental haplotypes and sex-specific recombination maps;
2. *Pilot simulations*
  For each pilot simulation 1..*MPILOT*:
  - Draw *s* and *h* from their prior distributions;
  - For each trio 1..*n*, compute the fitness of zygotes 1..150;
  - For each trio 1..*n*, sample one zygote with probability proportional to its fitness;
  - Compute summary statistics *Q*_sim_ for the sample on *n* selected zygotes;
3. *Updating priors*
  Accept a proportion *t* of the pilot simulations using the standard rejection algorithm based on the Euclidean distances between *Q*_obs_ and each *Q*_sim_;
  Set the sorted arrays of accepted *s* and *h* values as prior distributions for step 4;
4. *Final simulations*
  For each simulation 1..*MFINAL*:
  - Draw *s* and *h* from their updated prior distributions using Algorithm 2;
  - For each trio 1..*n*, compute the fitness of zygotes 1..150;
  - For each trio 1..*n*, sample one zygote with probability proportional to its fitness;
  - Compute summary statistics *Q*_sim_ on the sample on *n* selected zygotes;
5. Use TIDES output files as input for abc_adjust.R to paint the posterior distributions of *s* and *h* using rejection followed by regression adjustment.

### Algorithm 2

1. Let *w*_1_ be an element drawn uniformly at random from the sorted array of parameter values;
2. If *w*_1_ is the first element of the array, let *w*_2_ be the next element;
  Else if *w*_1_ is the last element of the array, let *w*_2_ be the previous element;
  Else set *w*_1_ and *w*_2_ as the previous and next elements in the list, respectively;
3. Draw a random number uniformly between *w*_1_ and *w*_2_.

### Simulation study

When benchmarking inference on individual SNPs, genomes for the test data sets were simulated within TIDES itself; each of the two haplotypes in each parent received the derived allele with 50% probability. Then, when joining females and males in couples, we avoided matches in which both parents were homozygous for the same allele. When benchmarking inference on a large set of candidate SNPs (e.g., all nonsynonymous mutations), the simulation of the test data set was more involved. Parental genomes were generated using SLiM 3.1 (Haller and Messer 2019). The sequences were 66.8 Mb in size, approximately the size of the human exonic coordinates obtained with Ensembl annotation in the biomaRt package (https://bioconductor.org/packages/release/bioc/html/biomaRt.html). Nonsynonymous sites were distributed according to exome coordinates provided for GRCh38 in Ensembl, after removing overlapping genes. The nonsynonymous mutation rate was set to 6.65 × 10^−9^ per site per generation, and we used sex-specific recombination maps from deCODE (Halldorsson et al. 2019). The nonsynonymous mutation rate is in the low end of the spectrum commonly adopted, meaning that our simulations are conservative with respect to the amount of standing deleterious diversity, and therefore, our simulation study is likewise conservative with respect to statistical power. The demography of the sample approximated the demography of Europeans (Gravel et al. 2011), where we omitted African populations for computational efficiency as well as increased the number of generations from 58,000 to 58,300 so that approximately 60,000 diploid individuals are sampled in present time. To allow reproducibility, we set the random seed of simulations in each evolutionary scenario to its corresponding replicate number (one to 10). All scripts necessary to reproduce the above procedures can be found in the GitHub repository (see Software availability).

The simulations described above were performed under the Wright–Fisher model that uses relative fitness among individuals and in which selection occurs in the mating stage of the life cycle. Because we focused on viability selection, we had SLiM output genomes from the parental generation exclusively. These were then input in TIDES, where they underwent viability selection followed by random pairing of females and males, and reproduction; finally, viability selection was imposed on the resulting embryos. These trios became the “observed” data in each of our simulated scenarios. To ensure that in all scenarios exactly 50,000 children are available after viability selection in the embryos (i.e., so we can directly relate statistical power to sample size), we generated 10 embryos per couple, subsequently down-sampling the number of surviving children to 50,000 at random. Because for some couples more than one child survives this process, our simulated data sets naturally contain siblings. Viability selection was executed according to the description in the SLiM manual (individuals were deleted if their fitness was smaller than a uniform random number between zero and one) and using the same *s* and *h* values as in the Wright–Fisher step of the simulation, except for simulated data sets for which the purpose was to test sensitivity to recent changes in *s* (Fig. 3). For the data sets in which the selection coefficient of each mutation follows a DFE, this information was extracted from the VCF files output by SLiM, and the fitness of each individual was computed using its exact genotype-fitness map. In summary, the simulated trios experienced a Wright–Fisher human demographic model with mating selection, followed by non-Wright–Fisher dynamics with viability selection in the last two generations.

For the simulations to assess the frequency of strongly deleterious variants segregating in the population (Table 1), we used a simpler demographic model in which the population size is constant at 10,000 individuals for 57,000 generations and then grows for 1000 generations until it reaches 10,000,000. In these simulations, the number of target sites is 1,000,000 (for *s* = −0.1), 5,000,000 (for *s* = −0.05), and 5,000,000 (for *s* = −0.01), with a constant recombination rate of 10^−3^ between each pair of sites in order to partially mimic their dispersion across the genome. Simulations to benchmark single-SNP inference were conducted within TIDES itself by assigning the derived allele to each parental haplotype according to a Bernoulli trial with *P* = 0.5. When performing inference on (single) beneficial SNPs, we subtracted 0.2 from the fitness of each individual throughout the ABC simulations to prevent individuals from having survival probabilities > 1.

Simulations of tight linkage blocks were again performed within TIDES itself. We simulated haplotypes with 11 SNPs, each separated by a 1-kb distance. The relative frequency of each SNP was drawn from a geometric distribution with mean equal to 0.5. The frequency of the middle SNP (at position 6) was set to exactly 0.5. The true selection coefficient of the middle SNP was set to −0.1, whereas the true selection coefficient of its neighbors was set to either −0.1 (model 1×) or −0.01 (model 10×). SNPs were then distributed among simulated haplotypes in maximum LD: Not only were all pairwise *D*′ values equal to 1.0, but we also maximized higher-order linkage among the SNPs. This is equivalent to there being a single genealogical tree describing the ancestry of the resulting haplotypes (in other words, zero recombination events in their past), with mutations on different branches of this tree determining their frequency. Haplotypes were then assembled into diploid individuals, and diploid individuals were randomly paired to form couples. Reproduction was performed with a uniform recombination rate of 10^−12^ per base-pair per generation during meiosis to keep the LD intact. Viability selection was performed based on the multiplicative fitness model that takes into consideration all SNPs in a diploid individual. Inference was performed by considering only the middle SNP. In other words, TIDES was blind to the effect of its neighbors in the tight linkage block (i.e., only one SNP was assumed to be potentially under selection when performing inference).

In our simulation study of human-like exons, we omitted de novo mutations (in the last generation) from our test data in order to focus on the reduction of deleterious variation caused by negative selection between parents and offspring. However, we provide two options for TIDES users to accommodate de novo mutations in real data sets. First, one can specify the total number of sites that can be targeted by deleterious mutations (*L*) as well as the mutation rate per site per generation among these sites (μ). In this case, SNPs are added to each simulated zygote with Poisson rate equal to *L* × μ. In case the user-specified rate is zero (its default value), de novo mutations are identified as those absent from parents but present in children, from which they are removed. These options are presented in the test run that can be found in TIDES GitHub repository (see Software availability).

### Software availability

The TIDES software package, composed of the simulation engine written in C++ and R scripts (R Core Team 2020) for downstream analyses and visualization of the results, is freely available as Supplemental Code and at GitHub (https://github.com/gvbarroso/TIDES).

## Supplementary Material

Supplemental Material
