# Supplementary material for: Inferring the mode and strength of ongoing selection
Source: Genome Res. 2023 Apr;33(4):632–43. doi: 10.1101/gr.276386.121 (PMC10234300; doi:10.1101/gr.276386.121)
Supplement: Supplemental Material [file supp_33_4_632__DC1.html]

Inferring the mode and strength of ongoing selection — Inferring the mode and strength of ongoing selection — Supplemental Material 

# Inferring the mode and strength of ongoing selection

## Supplemental Material

- Supplementary\_information.pdf
- Supplemental\_Code.zip
