# Supplementary material for: Inferring the mode and strength of ongoing selection
Source: Genome Res. 2023 Apr;33(4):632–43. doi: 10.1101/gr.276386.121 (PMC10234300; doi:10.1101/gr.276386.121)
Supplement: Supplemental Material [file supp_gr.276386.121_Supplementary_information.pdf]

## Supplemental Figures:

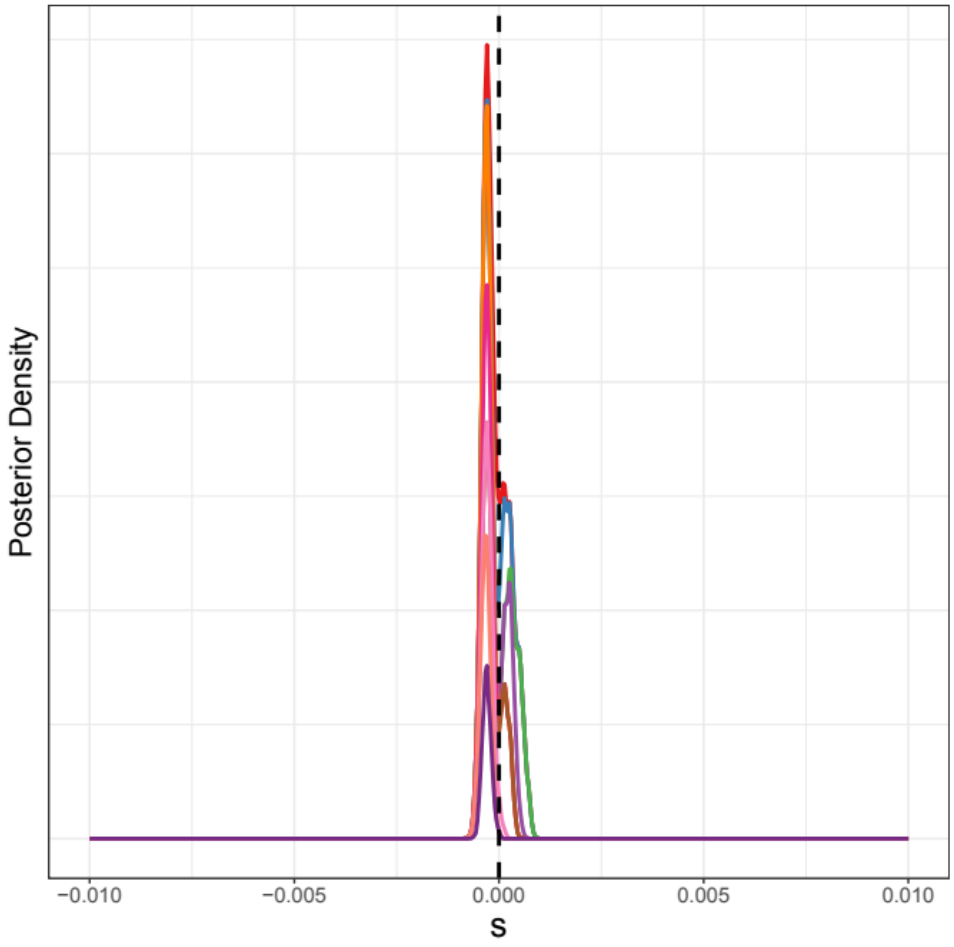


**Supplemental Figure S1:** Posterior distributions of *s* under neutrality. Each color represents the posterior density inferred from one of 10 replicate scenarios. Here 50,000 trios are used.

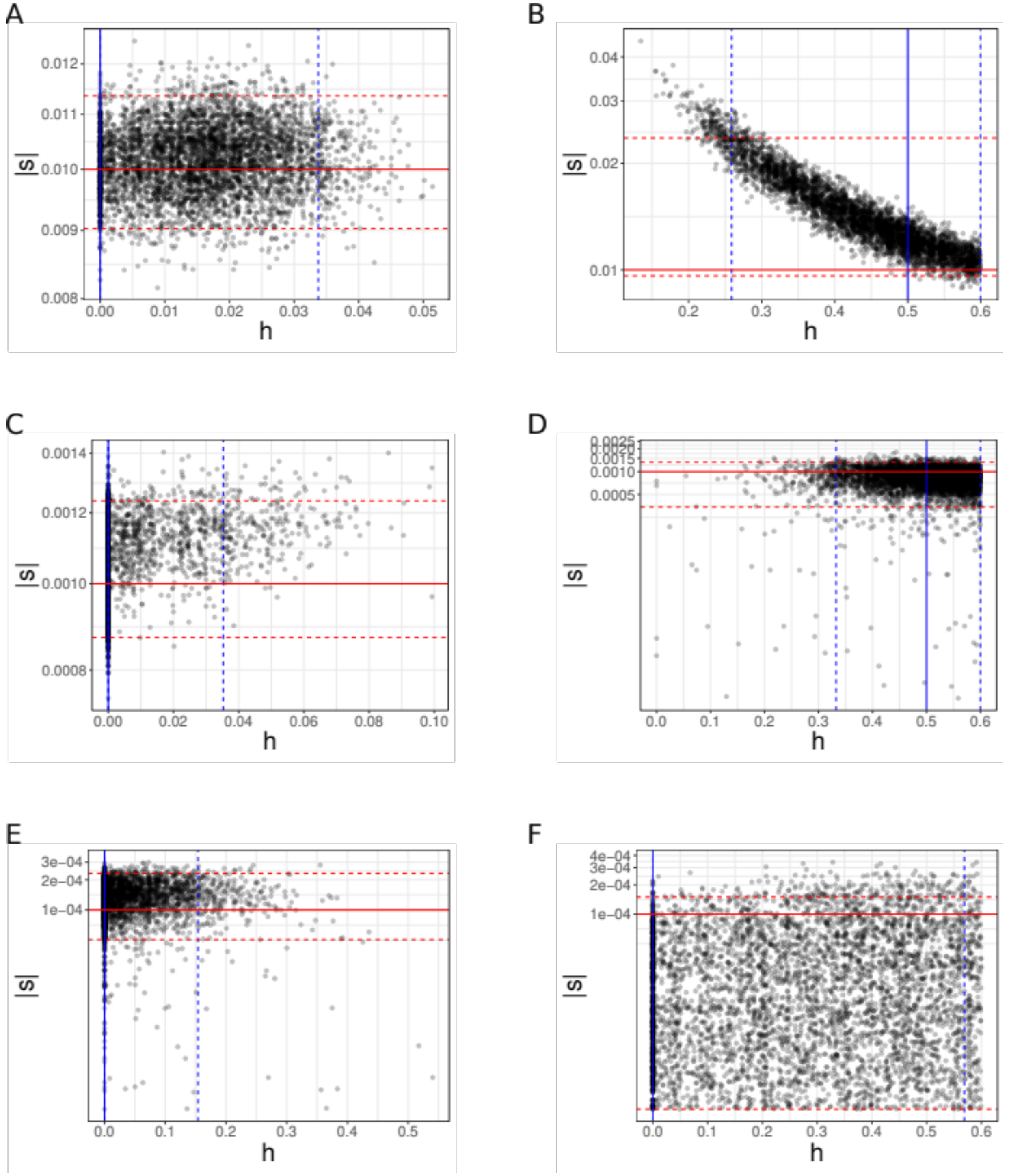


**Supplemental Figure S2:** Joint posterior distributions of *s* and *h* under different magnitudes of selection (**top:** *s* = -0.01; **mid**: *s* = -0.001; **bottom**: *s* = -0.0001) and dominance coefficients (**left:** *h* = 0; **right:** *h* = 0.5). In each plot there are 5,000 posterior samples, with true values of the simulated parameters shown by solid lines and the 95% CI of the posterior distributions shown by dashed lines (red for *s*, blue for *h*). One random replicate from each of the six scenarios is displayed. Here 50,000 trios are used.

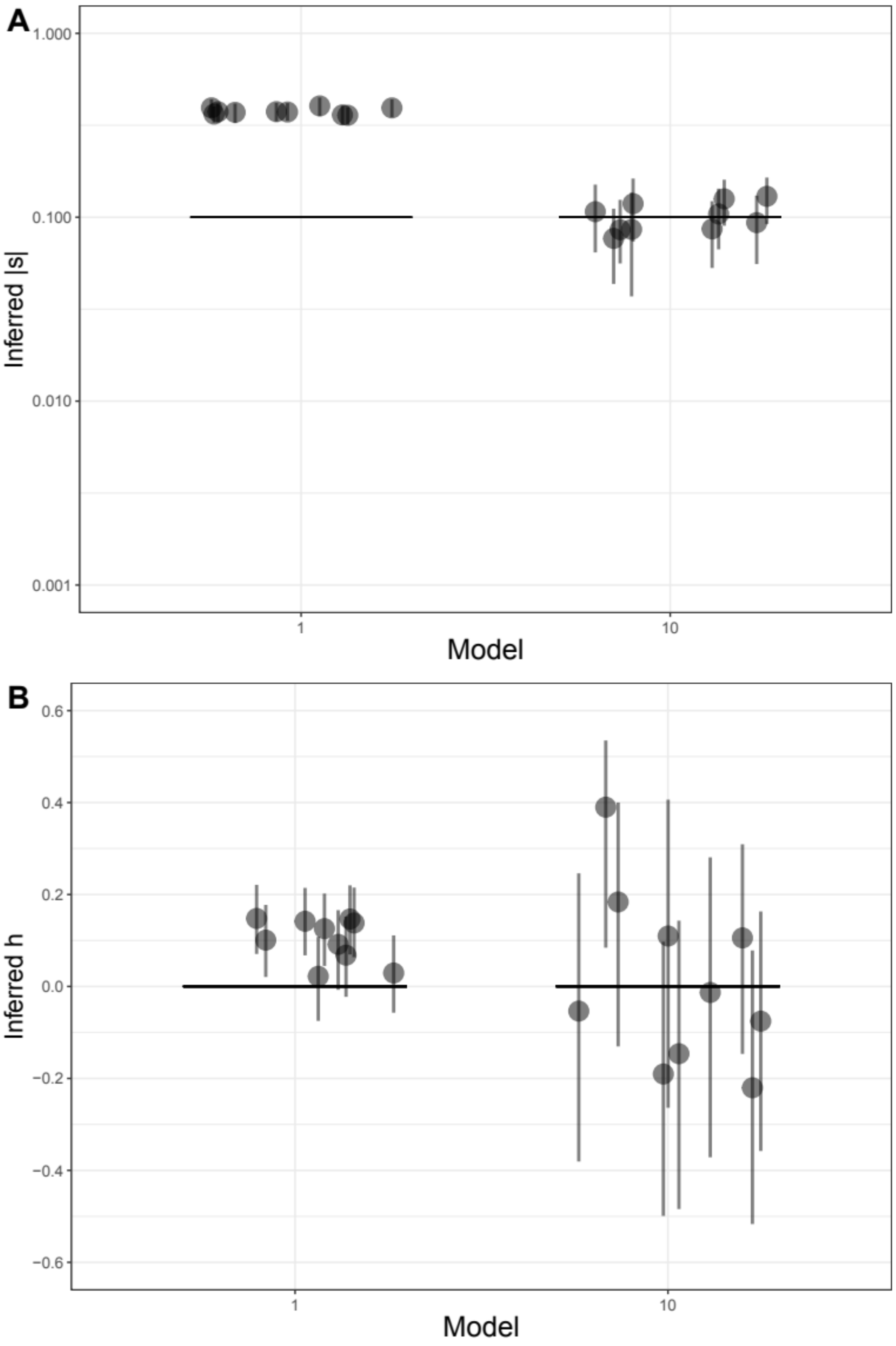


**Supplemental Figure S3:** Posterior distributions of A) *s* and B) *h* for a single SNP (10 replicates) that belongs to a tight linkage block with 10 neighbors (all pairwise D' equal to 1) that are ignored by the inference procedure. The focal SNP has frequency = 0.5 while the frequency of the others follows a geometric distribution with mean = 0.5. **Left:** negative selection against the focal SNP is as strong as against its neighbors. **Right:** negative selection against the focal SNP is 10 times stronger than against its neighbors. True values of the simulated parameters are shown as black horizontal segments, with medians of the inferred posterior distributions denoted by gray shapes and their 95% credible intervals by gray vertical lines. Here 10,000 trios are used.

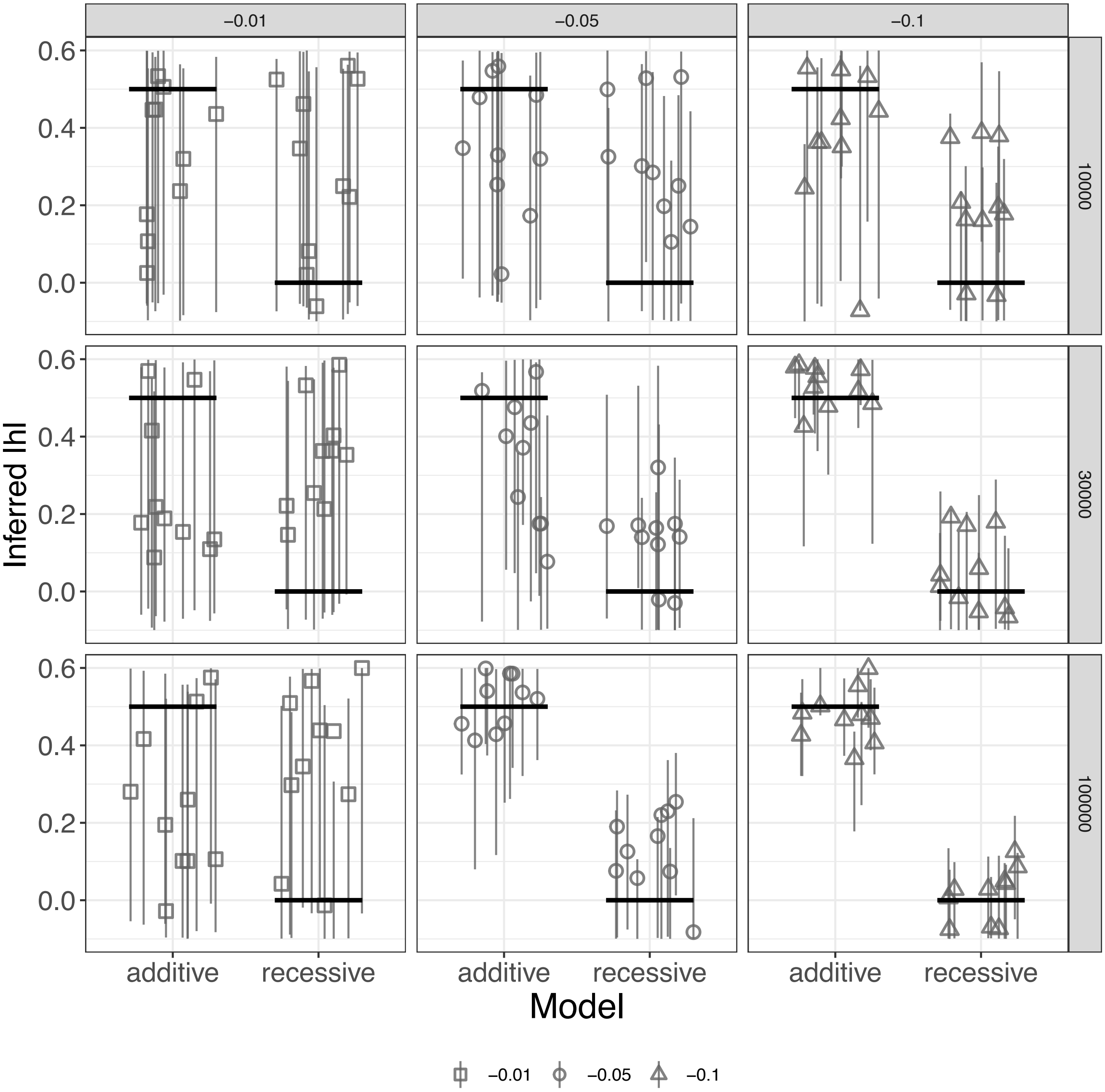


**Supplemental Figure S4:** Inference of *h* from a single deleterious SNP with different dominance effects. Each scenario includes the estimates from 10 simulated datasets. Columns show different values of the true selection coefficient, and rows show different sample sizes, in terms of the number of trios used. True values are shown as black horizontal segments, with medians of the inferred posterior distributions denoted by gray shapes and their 95% credible intervals by gray vertical lines.

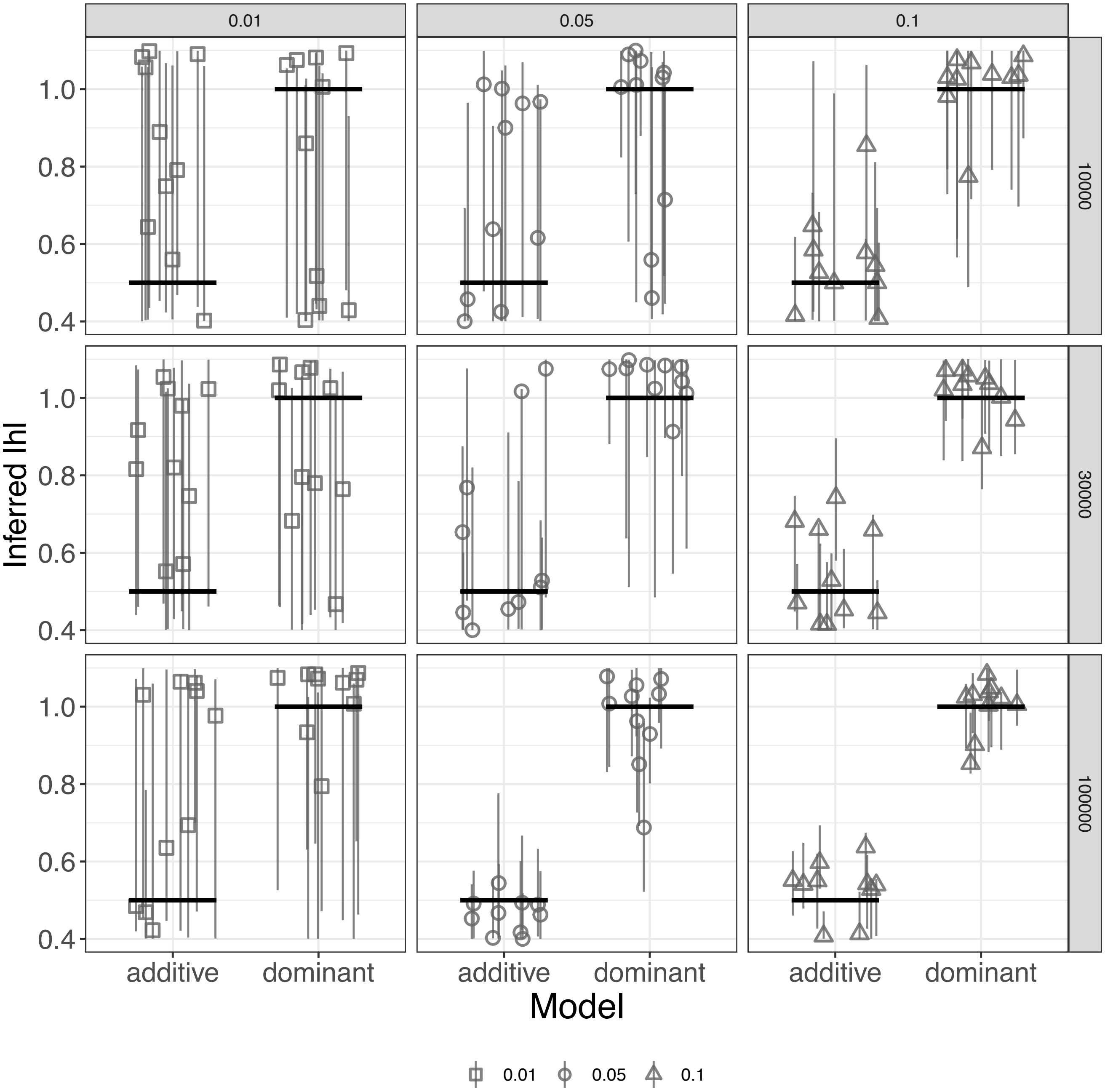


**Supplemental Figure S5:** Inference of *h* from a single beneficial SNP with different dominance effects. Each scenario includes the estimates from 10 simulated datasets. Columns show different values of the true selection coefficient, and rows show different sample sizes, in terms of the number of trios used. True values are shown as black horizontal segments, with medians of the inferred posterior distributions denoted by gray shapes and their 95% credible intervals by gray vertical lines.

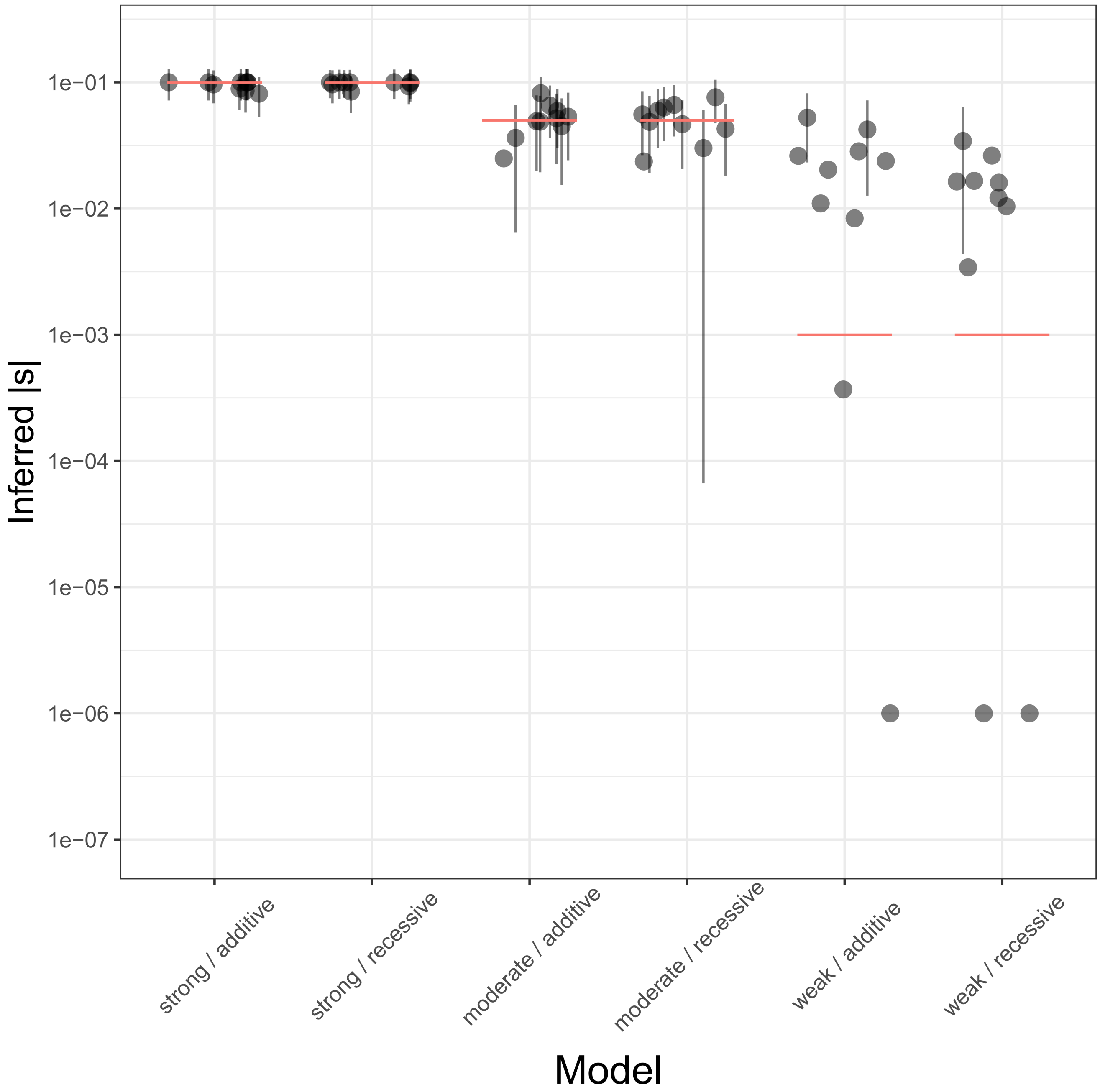


**Supplemental Figure S6:** Maximum likelihood inference of *s* for a single SNP (10 replicates) with average frequency equal to 0.5 among the family trios. True selection coefficients (in this setting, strong = -0.1, moderate = -0.05, weak = -0.01) are shown by the red horizontal bars. "Additive" and "recessive" models refer to *h* = 0.5 and *h* = 0, respectively. Vertical bars correspond to 95% confidence intervals (when absent, the optimization method could not estimate the standard error of the parameter). Here 50,000 trios are used.

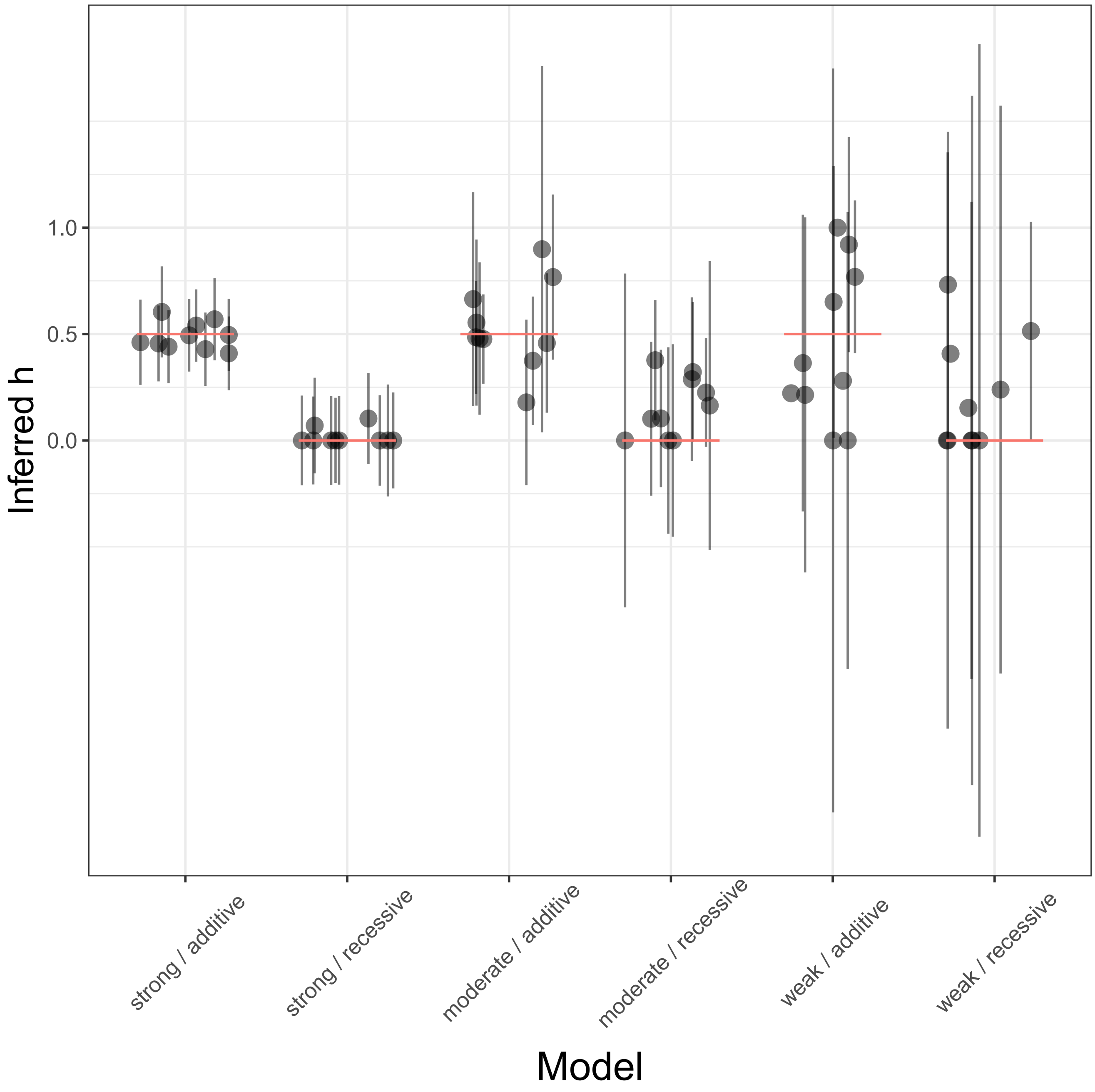


**Supplemental Figure S7:** Maximum likelihood inference of *h* for a single SNP (10 replicates) with average frequency equal to 0.5 among the family trios. True dominance coefficients ("additive", *h* = 0.5 and "recessive", *h* = 0) are shown by red horizontal bars. "Strong", "moderate" and "weak" models of selection as described in Supplemental Figure S6. Vertical bars correspond to 95% confidence intervals (when absent, the optimization method could not estimate the standard error of the parameter). Here 50,000 trios are used.

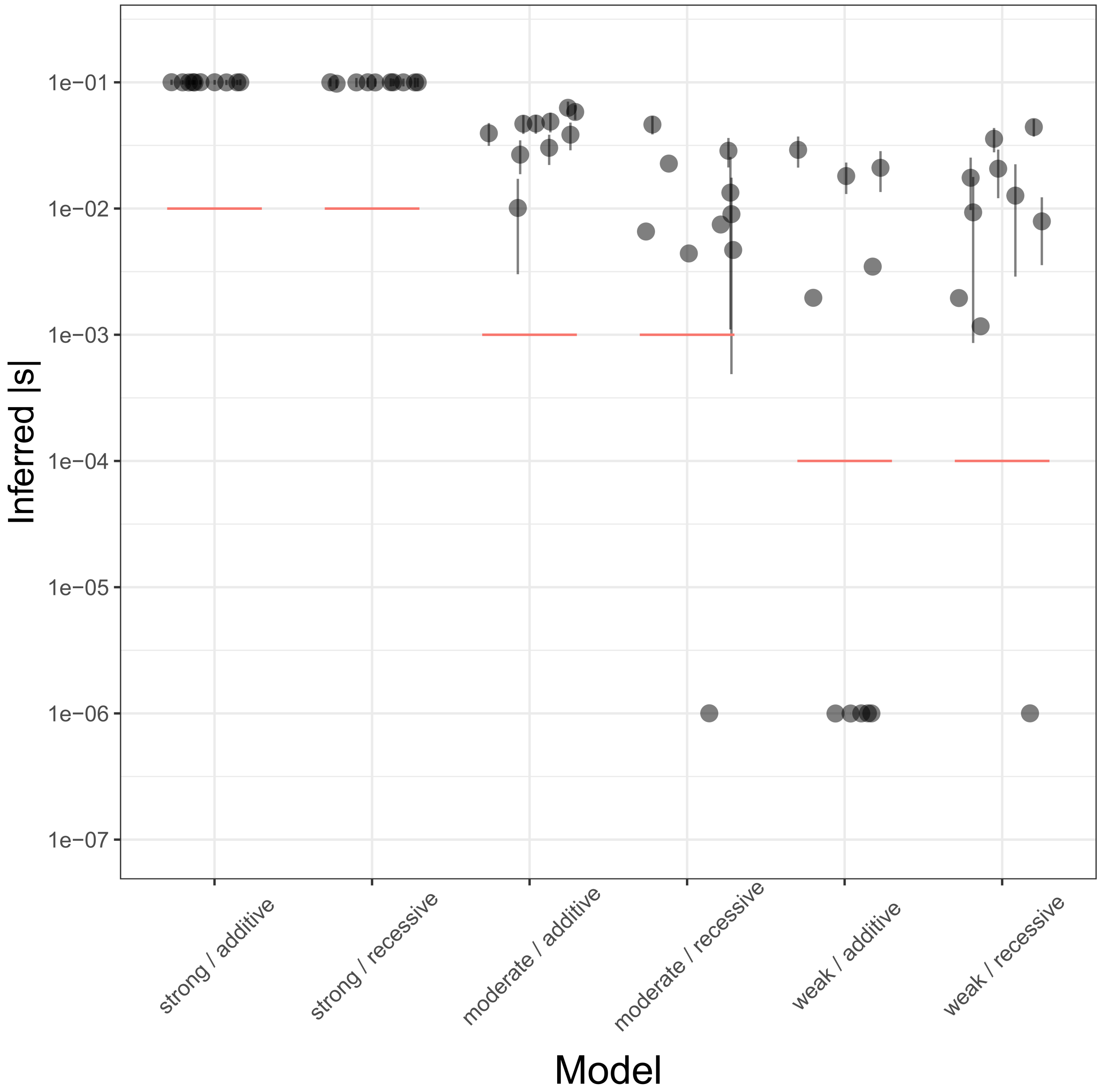


**Supplemental Figure S8:** Maximum likelihood inference of *s* for 128 linked SNP (10 replicates) with average frequency equal to 0.2 among the family trios. True selection coefficients (in this setting, strong = -0.01, moderate = -0.001, weak = -0.0001) are shown by the red horizontal bars. "Additive" and "recessive" models refer to $h = 0.5$ and $h = 0$, respectively. Vertical bars correspond to 95% confidence intervals (when absent, the optimization method could not estimate the standard error of the parameter). Here 50,000 trios are used.

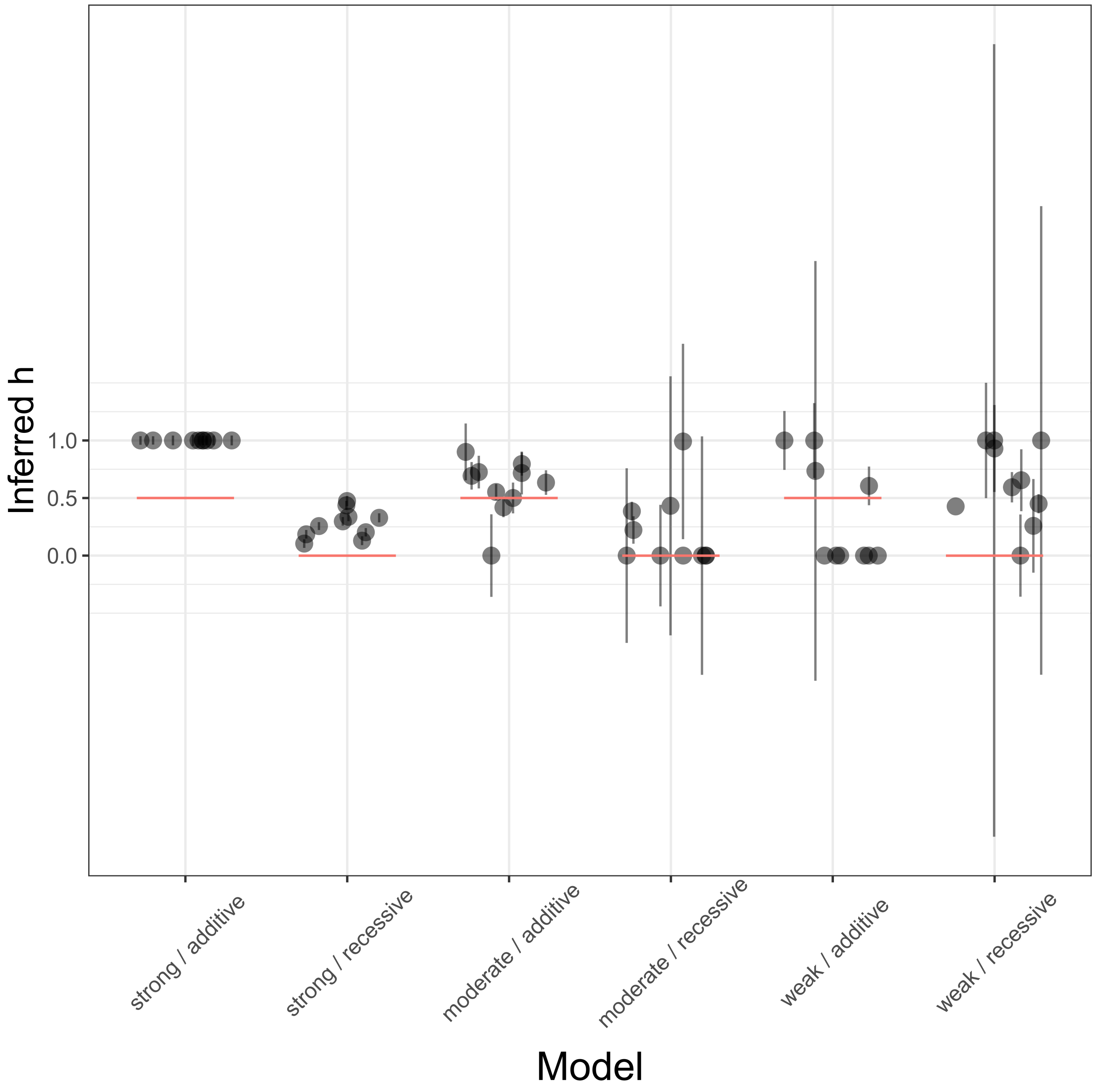


**Supplemental Figure S9:** Maximum likelihood inference of $h$ for 128 linked SNP (10 replicates) with average frequency equal to 0.2 among the family trios. True dominance coefficients ("additive", $h$ = 0.5 and "recessive", $h$ = 0) are shown by red horizontal bars. "Strong", "moderate" and "weak" models of selection as described in Supplemental Figure S8. Vertical bars correspond to 95% confidence intervals (when absent, the optimization method could not estimate the standard error of the parameter). Here 50,000 trios are used.

**Note S1: Comparing TIDES with Maximum Likelihood Estimates**

In this article we have presented a new model that uses ABC methodology to simultaneously infer *s* and *h* from transmission distortion patterns in family trios. The biggest advantage of ABC over likelihood-based methods is its flexibility, *i.e.*, the ability to study complex problems where a precise likelihood function cannot be specified. To demonstrate the importance of modeling LD to the inference of selection suing family trios, we developed a maximum-likelihood method to infer *s* and *h* and applied it to two scenarios that we have studied above: first, inference on single SNPs of frequency 0.5 in the dataset (*s* ranging from $-10^{-2}$ to $-10^{-1}$), for which the likelihood function is precise; second, inference on multiple SNPs simultaneously (in this example, 128 SNPs of average frequency 0.2 in the dataset, *s* ranging from $-10^{-4}$ to $-10^{-2}$), for which the likelihood function is biased because it assumes independence among SNPs (see below). The simulations to benchmark the maximum-likelihood method were also performed within TIDES, with 128 SNPs whose frequencies were drawn from a geometric distribution with mean equal to 0.2 and pairwise D' values drawn from a Beta distribution with mean equal to 0.045.

Like TIDES, our maximum-likelihood method considers informative trios (*ie*, those where the parental genotypes can produce more than one offspring genotype). Writing *A* for the ancestral allele and *a* for the derived (deleterious) allele, for any given locus there are three such configurations: (1) *AA x Aa,* (2) *Aa x Aa,* and (3) *Aa x aa.* We imagine sampling a dataset consisting of thousands of family trios, and let

$c_1$ be the count of *AA* genotypes born from *AA x Aa* parents;

$c_2$ be the count of *Aa* genotypes born from *AA x Aa* parents;

$c_3$ be the count of *AA* genotypes born from *Aa x Aa* parents;

$c_4$ be the count of *Aa* genotypes born from *Aa x Aa* parents;

$c_5$ be the count of *aa* genotypes born from *Aa x Aa* parents;

$c_6$ be the count of *AA* genotypes born from *Aa x aa* parents;

$c_7$ be the count of *Aa* genotypes born from *Aa x aa* parents.

The likelihood of observing a dataset defined by the counts $c_1$–$c_7$ follows an multinomial distribution:

$$P(D \vee M) = K \times \left(\frac{1}{(2 \times \bar{w_1})}\right)^{(c_1)} \times \left(\frac{(1 + h \times s)}{(2 \times \bar{w_1})}\right)^{(c_2)} \times \left(\frac{1}{(4 \times \bar{w_2})}\right)^{(c_3)} \times \left(\frac{(1 + h \times s)}{(2 \times \bar{w_2})}\right)^{(c_4)} \times \left(\frac{(1 + s)}{(4 \times \bar{w_2})}\right)^{(c_5)} \times \left(\frac{(1 + h \times s)}{(2 \times \bar{w_3})}\right)^{(c_6)} \times \left(\frac{(1 + s)}{(2 \times \bar{w_3})}\right)^{(c_7)}$$

where $\bar{w_1}$, $\bar{w_2}$ and $\bar{w_3}$ represent the average fitness of zygotes produced by the parental configurations 1, 2 and 3, respectively, and *K* is the normalizing constant giving the number of genotype permutations in the observed dataset, $K = \frac{(n!)}{(c_1! \times c_2! \times c_3 \times c_4 \times c_5 \times c_6 \times c_7)}$.

In words, the probability of the entire dataset is the probability of each (independent) genotype being born from its corresponding parental configuration. These, in turn, are given by the probability of their (Mendelian) transmissions, weighted by their fitnesses (1 for homozygous ancestral individuals, $1 + h \times s$ for heterozygotes and $1 + s$ for homozygous derived, see **equation 1**). Furthermore, because we are conditioning on the successful birth of the children present in the data (parents without children are not ascertained), we must divide each of the transmission probabilities by the average fitness of the offspring that could be generated by each couple so that we have a proper probability measure.

Applied to a single SNP of large effect, the maximum likelihood method yields accurate results for both *s* and *h* that are on par with those obtained with TIDES (**Supplemental Figure S6, S7**). However, when linked SNPs are considered simultaneously (see above), the estimates are heavily biased towards inferring stronger selection coefficients because linkage imposes a higher load than expected if the

deleterious mutations segregated independently (**Supplemental Figure S8**). In such cases, the point estimates of (average) *s* can be as low as -0.1, the minimum allowed by our numerical optimization procedure. In fact, the MLE is also biased towards inferring high *h* values (up to 1.0, the maximum allowed by our numerical optimization procedure) since in this case, the strong transmission distortion observed among the linked deleterious SNPs are better explained by the model of dominant mutations (**Supplemental Figure S9**). We have not observed such biases in TIDES (**Figure 2, 3, 5**), showcasing the flexibility of the ABC framework. The improved performance of TIEDES can be attributed to two factors. First, TIDES considers the average number of deleterious SNPs in both heterozygous and homozygous states, effectively combining them. Second, our simulation framework incorporates the process of meiotic recombination, modeling the change in LD between the parental and offspring generations. We conclude that TIDES better captures the essence of the family trio data structure better than the above likelihood-based method, and remains flexible to explore even more complex scenarios (see Discussion).
